# Thiol-SAM Concentration Effect on the Performance of Interdigitated Electrode-Based Redox-Free Biosensors

**DOI:** 10.3390/mi15101254

**Published:** 2024-10-12

**Authors:** Abdulaziz K. Assaifan

**Affiliations:** 1 Department of Biomedical Technology, College of Applied Medical Sciences, King Saud University, P.O. Box 10219, Riyadh 11433, Saudi Arabia; aassaifan@ksu.edu.sa; 2 Biological and Environmental Sensing Research Unit, King Abdullah Institute for Nanotechnology, King Saud University, P.O. Box 2455, Riyadh 11451, Saudi Arabia

**Keywords:** non-faradaic EIS, cysteamine self-assembled monolayer (SAM), gold interdigitated electrode (Au-IDE) biosensors, LDL-cholesterol detection

## Abstract

Despite the direct, redox-free and simple detection non-faradaic impedimetric biosensors offer, considerable optimizations are required to enhance their performance for the detection of various biomarkers. Non-faradaic EIS sensors’ performance depends on the interfacial capacitance between a polarized biosensor surface and the tested sample solution. Careful engineering and design of the interfacial capacitance is encouraged to magnify the redout signal upon bioreceptor–antigen interactions. One of the methods to achieve this goal is by optimizing the self-assembled monolayer concentration, which has not been reported for non-faradaic impedimetric sensors. Here, the impact of alkanethiolate (cysteamine) concentration on the performance of gold (Au) interdigitated electrode (Au-IDE) biosensors is reported. Six sets of biosensors were prepared, each with a different cysteamine concentration: 100 nM, 1 μM, 10 μM, 100 μM, 1 mM, and 10 mM. The biosensors were prepared for the direct detection of LDL cholesterol by attaching LDL antibodies on top of the cysteamine via a glutaraldehyde cross-linker. As the concentration of cysteamine increased from 100 nM to 100 μM, the sensitivity of the biosensor increased from 6.7 to 16.2 nF/ln (ng/mL). As the cysteamine concentration increased from 100 μM to 10 mM, the sensitivity deteriorated. The limit of detection (LoD) of the biosensor improved as the cysteamine increased from 100 nM to 100 μM (i.e., 400 ng/mL to 59 pg/mL). However, the LoD started to increase to 67 pg/mL and 16 ng/mL for 1 mM and 10 mM cysteamine concentrations, respectively. This shows that the cysteamine concentration has a detrimental effect on redox-free biosensors. The cysteamine layer has to be as thin as possible and uniformly cover the electrode surfaces to maximize positive readout signals and reduce negative signals, significantly improving both sensitivity and LoD.

## 1. Introduction

Over the years, electrochemical impedance spectroscopy (EIS) has been utilized in a wide range of applications, including batteries [1], fuel cells [2], corrosion studies, and biosensing [3,4]. Owing to its low cost, it is an attractive technique for biosensing applications. EIS-based biosensors can be fabricated on flexible substrates, which makes them great candidates as wearable biosensors in the future [5]. In addition, they operate at small AC potential (typically 10 mV) and they can be fabricated using low-cost and mass-production techniques such as roll-to-roll printing methods [6]. In this regard, EIS biosensors are desirable for the mass screening of diseases at low cost, allowing early medical intervention and reducing any health implications in the case of an epidemic/pandemic. EIS is divided into two modes, faradaic and non-faradaic. The first has been widely used for biosensing applications for the detection of various pathogens and diseases [7,8,9]. It is based on charge transfer resistance (R_ct_) alterations at the biosensor surface due to bioreceptor–biomarker interactions. Despite the outstanding biosensing performances of faradaic impedimetric biosensors, they require the use of redox probes such as ferro/ferricyanide ([Fe(CN)_6_]3^−^/4^−^), responsible for reduction/oxidation reactions at the biosensor surface [10]. These redox probes add an extra step, which increases the time spent on detection. In addition, redox probes are involved with toxicity, which can be harmful to health practitioners and patients [10]. Moreover, reference electrodes are required when running faradaic EIS measurements, which also adds an extra step to fabrication and contributes to material waste.

**Figure 1:**
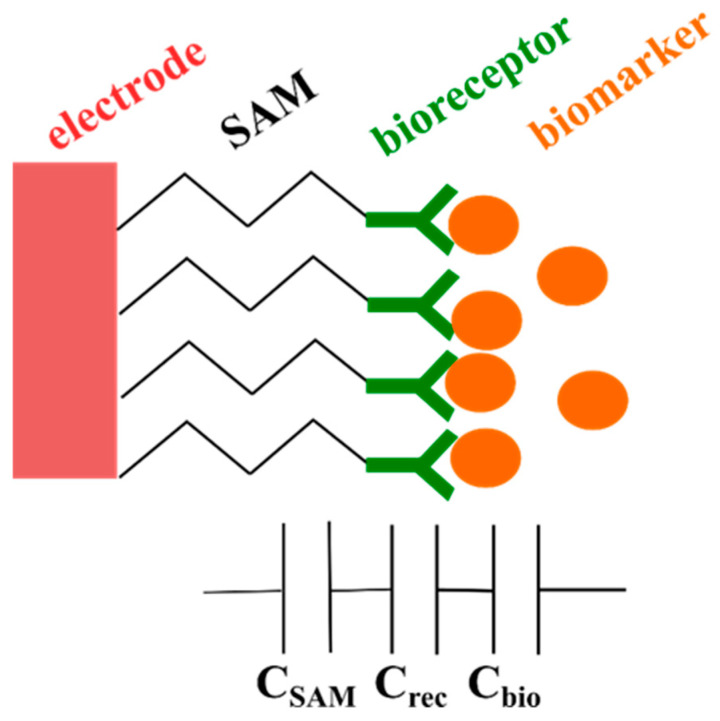
Non-faradaic biosensor structure.

On the contrary, reference electrodes are not required in non-faradaic EIS measurements, which reduces the size and subsequently the cost involved in their fabrication processes. Non-faradaic impedimetric devices do not need redox probes. This feature offered by non-faradaic impedimetric biosensors offers on-site, label-free and direct detection of biomarkers. Transduction is based on interfacial capacitance alterations when the bioreceptors interact with the biomarkers. This interfacial capacitance is induced when a small AC voltage is applied at the electrode, which causes the arrangement of oppositely charged ions at the biosensor–sample interface [11]. This interfacial capacitance consists of a number of capacitances arranged in series. These capacitances are a self-assembled monolayer (SAM) (or cross-linking layer) capacitance (C_SAM_), bioreceptor layer capacitance (C_rec_), and bioreceptor/biomarker capacitance (C_bio_). Figure 1 shows the components that make up non-faradaic biosensors. Changes in interfacial capacitance in non-faradaic impedimetric biosensors can be due to changes in biosensor surface area upon biomarker attachment to bioreceptors, change in dipole orientation, thickness increase, or dielectric constant alterations [12].

To magnify the change in capacitance for C_bio_, C_SAM_ and C_rec_ must be as large as possible. This can be accomplished by passivating the electrode with a thin and dense layer that is fully covering the electrode surface and prevents the transfer of ions from sample bulk to electrode surface [13]. This is critical for the performance of non-faradaic impedimetric biosensors. Careful engineering and design of the cross-linking layers must be carried out to enhance redox-free detection.

In the past, different non-faradaic sensors consisting of different cross-linking layers such as APTES [6], cysteamine [14], DSP [15] and 11-mercaptoundecanoic acid (11-MUA) [16] were used for the detection of various biomarkers. However, the cross-linking layer concentration, which is crucial for biosensors’ detection properties, was not investigated in the context of non-faradaic impedimetric biosensors. Optimizing the cross-linking layer concentration would enhance biosensing performances such as sensitivity and LoD. In this regard, and to the best of the author’s knowledge, the effect of cysteamine concentration on the performance of non-faradaic impedimetric biosensors is here reported for the first time. Six different sets of biosensors consisting of interdigitated electrodes made of gold (Au-IDEs) and modified with different cysteamine concentrations (i.e., 100 nM, 1 μM, 10 μM, 100 μM, 1 mM, and 10 mM) were prepared and used for non-faradaic biosensing of low-density lipoprotein (LDL). Firstly, the electronic properties of cysteamine-functionalized Au-IDEs were investigated through faradaic EIS. Then, glutaraldehyde was attached to the amine groups of cysteamine layers followed by LDL-antibody immobilization for the non-faradaic measurements of the LDL antigens. The sensitivity and limit of detection for each biosensor was calculated. The ability to optimize the cysteamine layer concentration for non-faradaic EIS measurements enhanced their performance and improve the signal-to-noise ratio and eventually the overall biosensor performance.

## 2. Materials and Methods

### 2.1. Materials

Interdigital electrodes of gold (Au-IDEs) consisting of 100 µm-wide fingers and spacing between fingers of 100 µm were used for the detection of LDL. These electrodes were purchased from Metrohm Dropsense, Asturias, Spain. Cysteamine and glutaraldehyde (2.5% in H_2_O) were procured from Sigma Aldrich, Burlington, MA, USA. Bovine serum albumin (BSA) was obtained from the same supplier. Potassium ferricyanide (III) (K_3_Fe(CN)_6_) and potassium hexacyanoferrate(II) trihydrate (K_4_Fe(CN)_6_ 3H_2_O) were obtained from Sigma Aldrich, USA. Ethanol (99%) and phosphate-buffered saline (PBS) (pH of 7.4) were acquired from Fisher, Loughborough, UK. LDL antibody and LDL antigen were purchased from Sino Biological, Beijing, China. For selectivity experiments, the PP65 antigen of HCMV was purchased from Virusys, Milford, MA, USA. PDL1-antigen was purchased from Moleqcule-On, Auckland New Zealand and IL-8-antigen was supplied by Abcam, Cambridge, UK.

### 2.2. Electrochemical Characterization of Cysteamine-Functionalized Au-IDEs

**Figure 2:**
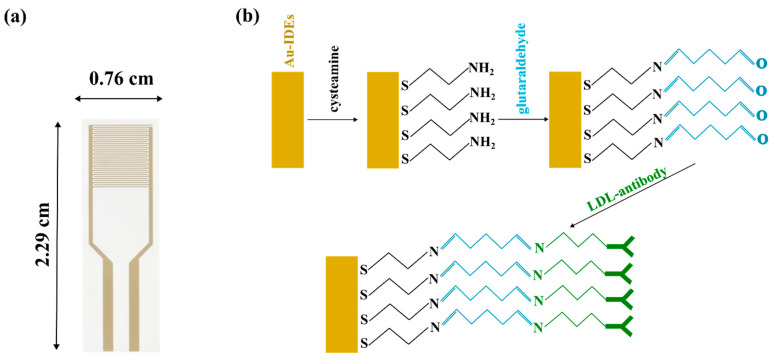
(**a**) Au-IDEs on white plastic substrate. (**b**) Biofunctionalization steps.

**Figure 3:**
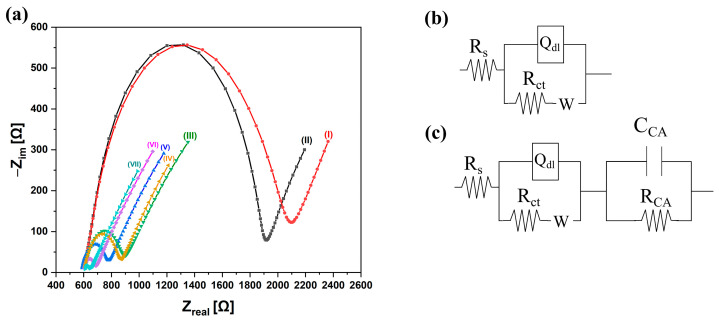
(**a**) Nyquist plot for (I) bare Au-IDEs and Au-IDEs functionalized with (II) 100 nM, (III) 1 µM, (IV) 10 µM, (V) 100 µM, (VI) 1 mM and (VII) 10 mM of cysteamine. (**b**) Randle circuit for bare Au-IDEs. (**c**) Randle circuit for cysteamine-functionalized Au-IDEs.

Six ethanolic solutions with different cysteamine concentrations were prepared, namely, 100 nM, 1 µM. 10 µM, 100 µM, 1 mM, and 10 mM. Cysteamine functionalization at the surface of the interdigitated electrodes (sensing window) (see Figure 2a) was carried out for one hour. Bare interdigitated electrodes and cysteamine-functionalized Au-IDEs were electrochemically characterized by faradaic EIS utilizing a potentiostat ((interface 1000™), GAMRY, Warminster, PA, USA) to determine the influence of cysteamine content on the impedimetric parameters and to justify the biosensing results. Impedimetric measurements were carried out in the presence of 50 µL of 5.0 mM K_3_Fe(CN)_6_/K_4_Fe(CN)_6_ (1:1 mixture) in PBS. Measurements were carried out under an applied potential of 10 mV between 200 mHz and 100 kHz. Randle circuits depicted from previous research (see Figure 3b,c) were used to extract the impedimetric parameters such as resistance of the solution (R_s_), resistance of the charge transfer (R_ct_), electrode resistance (R_Au-IDEs_), electrode capacitance (C_Au-IDEs_), cysteamine layer resistance (R_CA_), cysteamine layer capacitance (C_CA_), Warburg impedance (W), and constant phase element (Q_dl_) [17,18].

### 2.3. Biofunctionalization

As mentioned above, cysteamine was functionalized at the biosensor surface. After cysteamine incubation, ethanol was used to wash the surface followed by drying with air. The sensing window was then incubated with 50 μL of 2.5% of glutaraldehyde for one hour. The biosensor was then rinsed with DI water and blow-dried. Glutaraldehyde was prepared in PBS solution. Aldehyde groups of the glutaraldehyde covalently bind to the amine groups of the cysteamine. The amine groups of the antibodies then bind to the exposed aldehyde groups of the glutaraldehyde. LDL antibody (1 µg/mL) in 50 µL DI water was prepared and introduced at the sensing window for one hour. DI water was then used to clean the sensor surface. At the end of biofunctionalization, BSA was immobilized at active window by incubating 50 µL of 5% BSA in PBS for 30 min. Then, cleaning with DI water was undertaken with air-drying. Figure 2b illustrates the functionalization approach conducted.

### 2.4. Non-Faradaic EIS Measurements

After biofunctionalization, each biosensor was connected to the potentiostat. Initially, 50 µL PBS was placed at the sensor window to obtain the baseline readout and to allow time for biosensor surface conditioning [19]. Afterward, rinsing with DI water was performed at the sensing window to be incubated with five different LDL antigen concentrations (i.e., 3 ng/mL, 30 ng/mL, 300 ng/mL, 3 µg/mL and 30 µg/mL). Measurements using non-faradaic mode were then carried out after each LDL antigen concentration incubation and normalized to the baseline. Each incubation lasted for 5 min and 10 mV was the applied AC voltage in a frequency range of 100 kHz to 200 mHz. Open circuit potential measurement was undertaken before each EIS scan to ensure the stability of the biosensor. Likewise, non-faradaic experiments were undertaken to investigate the influence of blank samples (i.e., PBS). Each experiment was performed in triplicate. The sensitivity and LoD for each biosensor were calculated and compared with the other biosensors. Selectivity experiments were also carried out on two samples, each containing a mixture of different antigens, one with an LDL antigen and the other without.

## 3. Results and Discussion

Figure 3a illustrates the Nyquist plots for bare interdigital electrodes and cysteamine-functionalized interdigital electrodes. Faradaic EIS is a useful tool to investigate the surface and interface properties. The semicircles represent the charge transfer resistance, whereas the straight line is due to diffusion of molecules and ions from bulk solution to the Au-IDE surface. Figure 3b,c are the Randle circuits for bare interdigital electrodes and cysteamine-functionalized interdigital electrodes, respectively. Table 1 lists the impedimetric parameters for cysteamine-functionalized Au-IDEs. Initially, the impedimetric parameters of bare Au-IDEs were extracted. The bare Au-IDE capacitance (C_Au-IDEs_) and resistance (R_Au-IDEs_) was 7 nF and 1.5 Ω, respectively. Functionalizing the Au-IDE surface with cysteamine resulted in a decrease in charge transfer resistance (R_ct_). As the concentration of the functionalized cysteamine increased from 100 nM to 10 mM, the Rct decreased from 1388 to 39 Ω. This is attributable to the existence of a large number of cysteamine polarizable amine groups, which attract oppositely charged redox molecules to the Au-IDE surface and hence decrease charge transfer at the surface [20]. The continuous decrease in Rct with increasing cysteamine concentration shows that denser cysteamine molecules are available at the biosensor surface, which leads to more polarized amine groups at the surface of Au-IDEs. Furthermore, the constant phase element (Q_dl_), which represents the double-layer capacitive behavior of the system, decreased with increased cysteamine concentration. The *n* value shown in Table 1 indicates that the roughness of the cross-linking layer decreased with increased cysteamine concentration, which suggests that for high cysteamine concentrations, the surface becomes uneven and rough due to the formation of thick, stacked, and randomly oriented layers of cysteamine [21]. The decrease in Q_dl_ and its corresponding *n* value suggests that it is due to the increased thickness of the cysteamine layer at the Au-IDE surface. Similarly, the cysteamine layer capacitance C_CA_ lowered as the concentration was raised. This is a result of increased thickness of cysteamine [22]. The cysteamine layer resistance R_CA_ increased with increased cysteamine concentration. This is also attributed to more area being covered by cysteamine with a higher concentration, which contributed to an increase in resistance [18].

**Figure 4:**
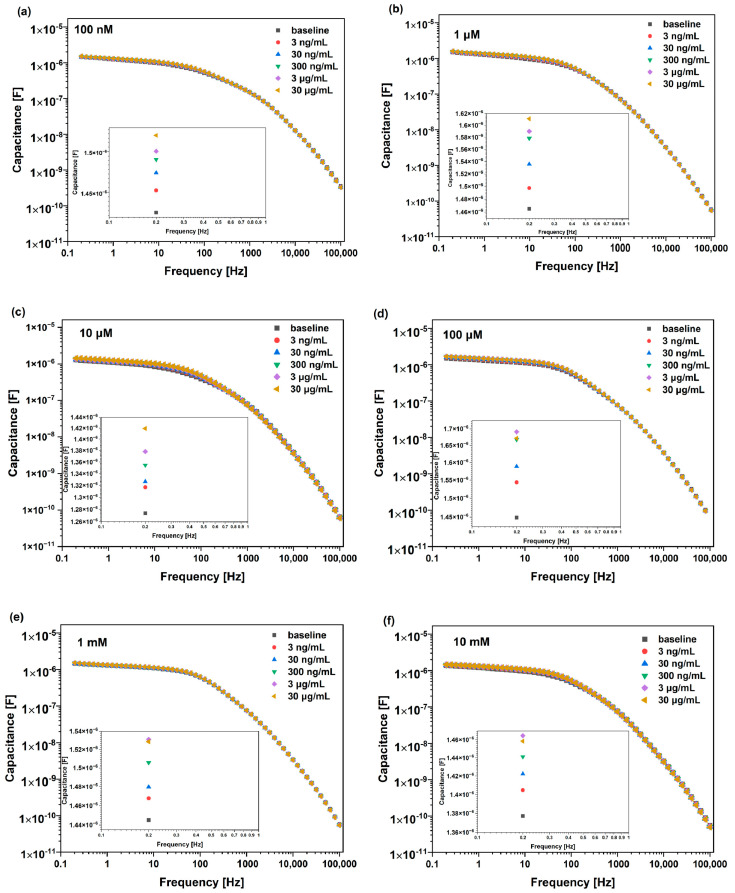
Typical Bode plots for Au-IDE biosensors functionalized with (**a**) 100 nM, (**b**) 1 µM, (**c**) 10 µM, (**d**) 100 µM, (**e**) 1 mM and (**f**) 10 mM of cysteamine.

**Figure 5:**
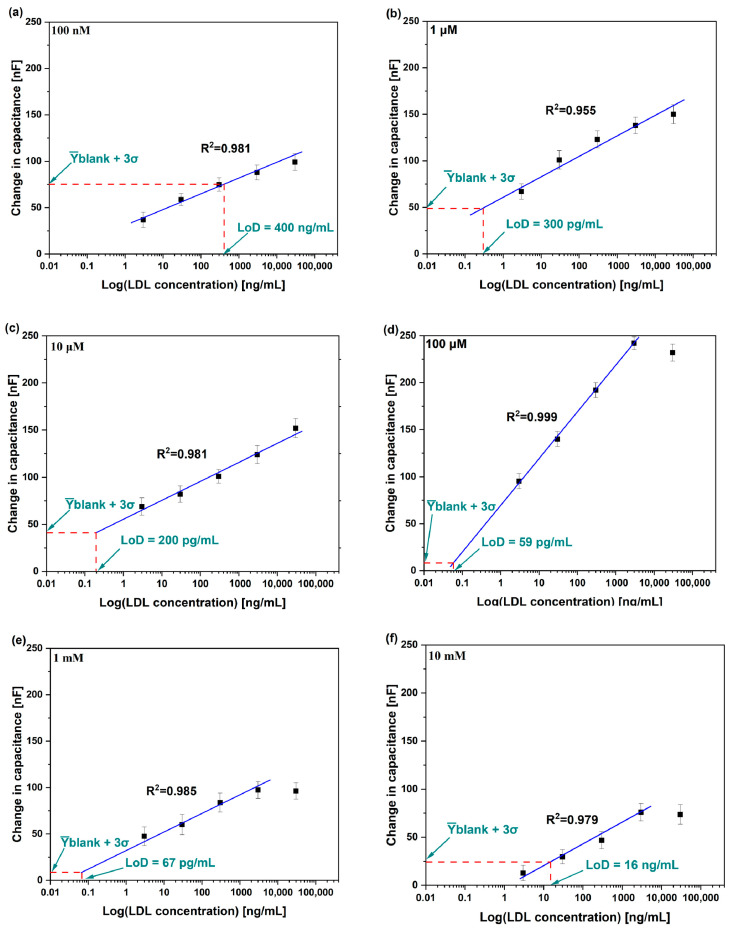
Calibration curves for Au-IDE biosensors functionalized with (**a**) 100 nM, (**b**) 1 µM, (**c**) 10 µM, (**d**) 100 µM, (**e**) 1 mM and (**f**) 10 mM of cysteamine.

Open circuit potential (OCP) was measured before achieving the Bode plots shown in Figure 4. Non-faradaic measurements were taken between 100 kHz and 200 mHz with an applied AC voltage of 10 mV. Figure 4 illustrates typical Bode plots for the Au-IDE biosensors tested against different concentrations of LDL antigen. Firstly, baseline measurement was undertaken. Then, the sensing windows were rinsed with DI water and incubated for 5 min with 50 µL of LDL antigen in PBS followed by EIS measurement. Five different concentrations of LDL were tested (i.e., 3, 30, 300, 3000 and 30,000 ng/mL) and DI water was used to clean the sensor after each tested concentration. Changes in capacitance reach their maximum at 200 mHz. Extracted data against LDL antigen were normalized to the baseline measurement. It is worth noting that changes at such low frequency are associated with bioreceptor–biomarker interaction at the biosensor surface. In future, a diagnostic device can be manufactured to operate at this single frequency, which would reduce the time required for detection. Detection at such low frequency is promising for the fabrication of economic, miniaturized, and mass-producible diagnostic devices. These diagnostic devices can be used by any individual, which allows mass screening and early medical intervention to prevent the spread of diseases. The capacitance increased for all sensors as the antigen concentration increased. The changes in capacitances when biosensors were tested against different LDL antigen concentrations were extracted and used to plot the calibration curve shown in Figure 5. Likewise, the six Au-IDE biosensors were tested against successive incubations of blank (PBS) samples to investigate the biosensors’ stability and to achieve the limit of detection (LoD).

**Figure 6:**
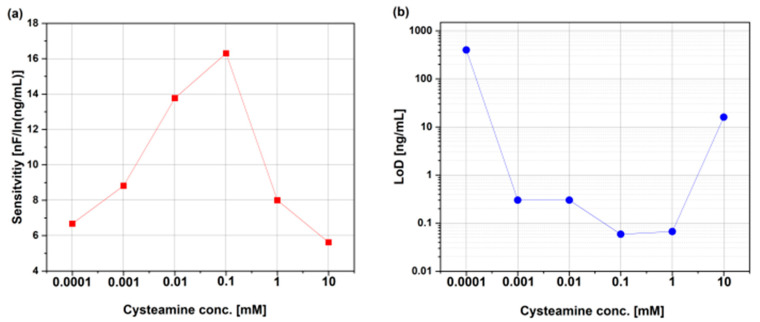
Effect of cysteamine concentration on (**a**) sensitivity and (**b**) LoD of Au-IDE biosensors.

Figure 5 shows calibration curves of each Au-IDE biosensor. The calibration curve consists of a solid blue line, which represents the linear fitting. The sensitivity for each biosensor was obtained from the slope. As the cysteamine concentration increased from 100 nM to 100 µM, the sensitivity of the biosensors increased. The sensitivity was 6.7, 8.7, 13.9 and 16.2 nF/ln (ng/mL) for the biosensors functionalized with 100 nM, 1 µM, 10 µM and 100 µM cysteamine concentrations, respectively. As the cysteamine concentration increased to 1 mM and 10 mM, the sensitivity dropped to 8 and 5.8 nF/ln (ng/mL), respectively (see Figure 6a). The increase in the cysteamine concentration from 100 nM to 100 µM resulted in an increase in the maximum change in capacitance (∆C) from approximately 100 to 240 nF. Increasing the cysteamine concentration at the Au-IDE biosensors surface resulted in more amine groups at the Au-IDE surface (i.e., more binding sites) for the antibodies at the surface and hence more binding sites for the antigens to bind to and eventually resulting in larger changes in capacitance. The drop in sensitivity for Au-IDE biosensors functionalized with 1 mM and 10 mM could be attributed to the formation of a thick cysteamine layer that passivated the Au-IDE electrodes from screening bioreceptor–biomarker interactions at the surface. It is worth noting that biosensor response for 100 μM, 1 mM and 10 mM showed saturation behavior at LDL antigen concentration of 30 μg/mL. This can be attributed to the passivation of the biosensor surface with large concentrations of cysteamine, which results in saturation in the biosensor performance.

The limit of detection was also calculated for each biosensor. LoD calculations were achieved graphically by finding the intersection between the linear line (blue solid line) and $\overset{-}{y}$blank + 3σ value [23,24], where $\overset{-}{y}$blank is the average of the change in capacitance when testing the biosensors against the blank (PBS) samples and σ is the average of the standard deviations of the change in capacitance when testing the blank samples. It was interesting to find that $\overset{-}{y}$blank + 3σ decreased with increased cysteamine concentration from 100 nM to 100 µM (see Figure 6b). This resulted in the LoD decreasing from 400 ng/mL to 59 pg/mL (see Figure 6b). This can be attributed to the insulating and thin cysteamine layer that prevented the travel of ions in PBS to the biosensor surface and hence reduced the value of $\overset{-}{y}$blank + 3σ and subsequently enhanced the LoD of the biosensors. On the other hand, as the cysteamine concentration increased to 1 mM and 10 mM, $\overset{-}{y}$blank + 3σ increased, which increased the LoD to 67 pg/mL and 16 ng/mL, respectively (see Figure 6b). Such high cysteamine concentrations may have resulted in an agglomeration of cysteamine and physically bound molecules at the biosensor surface that caused distribution to the non-faradaic biosensor properties. From the results obtained in this study, 100 µM cysteamine concentration is the optimum concentration to achieve the best sensitivity and LoD. However, saturation was observed when biosensors functionalized with 100 μM cysteamine were tested against 30 μg/mL of LDL antigen. On the other hand, biosensors functionalized with 10 μM cysteamine possess better linear range. Considering linear range, sensitivity, and LoD, biosensors functionalized with 10 μM of cysteamine are the optimal choice. The increase in cysteamine concentration from 100 nM to 100 µM reduced pinholes by forming a dense SAM layer at the biosensor surface (reflected by the extracted cysteamine layer resistance increase shown in Table 1) and hence improved the sensitivity and LoD of the biosensors. Increasing the cysteamine concentration to 1 mM and 10 mM drastically decreased the sensitivity due to the thick cysteamine layer, which prevented scanning surface interactions by the transducer.

**Figure 7:**
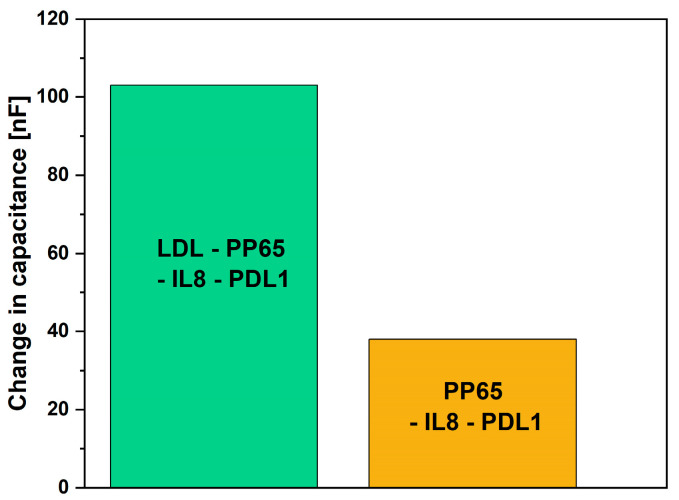
Selectivity behavior of the Au-IDE biosensor.

Selectivity of the biosensor functionalized with 10 µM was investigated. The biosensor was tested against two samples. The first consisted of LDL antigen, pp65 antigen, IL-8 antigen and PDL1 antigen. The other consisted of all those except LDL antigen. The concentration of each antigen was around 300 ng/mL. The change in capacitance when testing the biosensor against the sample that contained LDL antigen was 103 nF, whereas the change in capacitance when testing the biosensor against the sample that did not contain LDL antigen was 38 nF (see Figure 7). This demonstrates the selectivity of the biosensor in a sample that consists of different antigens.

## 4. Conclusions

In this work, the cysteamine cross-linking layer concentration effect on the performance of redox-free impedimetric biosensors was investigated. The optimal concentration was found to be 10 μM. Biosensors at this concentration demonstrated better biosensing performances against LDL antigen. Since the non-faradaic impedimetric biosensors’ performances largely depend on the physical properties of the cross-linking layer, it is strongly recommended to investigate and optimize different cross-linking layers concentrations on different electrode materials for redox-free (or non-faradaic) impedimetric detection of different biomarkers. The cross-linking layer must be impermeable to ions in the tested sample solution and must be thin enough to enhance the biosensor sensitivity through the enlarged electric potential due to the thin cross-linking layer, which does not passivate the potential across the bioreceptor–biomarker interface.

## Figures and Tables

**Table 1:** Extracted impedimetric parameters for bare Au-IDEs and cysteamine (CA)-functionalized Au-IDEs.

	R_s_ (Ω)	R_ct_ (Ω)	Q_dl_ (µF)	*n*	W (µΩS^−1^)	C_CA_ (nF)	R_CA_ (Ω)
Bare Au	604	1388	2.062	0.895	367		
100 nM CA	601	1305	1.97	0.824	325	281	31
1 µM CA	598	285	1.63	0.502	183	273	50.1
10 µM CA	605	261	1.48	0.43	244	266	67
100 µM CA	599	205	1.24	0.411	221	260	157
1 mM CA	605	82	1.12	0.37	252	243	201
10 mM CA	609	39	0.423	0.35	260	218	211

## Data Availability

Data are contained within the article.
